# Assessment of Bone Health Awareness and Education in Breast Cancer Patients with Bone Metastasis in the USA

**DOI:** 10.1007/s13187-023-02293-w

**Published:** 2023-04-28

**Authors:** Darcy R. Flora, Jennifer Schenfeld, Hossam Saad, Ben Cadieux, Guy Boike, Kimberly A. Lowe

**Affiliations:** 1 GRYT Health Inc., Rochester, NY USA; 2 Amgen Inc., Thousand Oaks, CA USA; 3 McLaren Bay Region Medical Center, Bay City, MI USA

**Keywords:** Breast cancer, Bone metastasis, Skeletal-related event, Bone targeting agent

## Abstract

Bone metastases are common in advanced breast cancer (BC) patients and increase the risk for skeletal-related events (SREs), which present a significant health and economic burden. Bone targeting agents (BTAs) can improve health-related quality of life by delaying or preventing SREs; nevertheless, a significant portion of eligible BC patients are not receiving this therapy. A bone health education needs assessment survey was conducted to examine cancer-related bone health awareness and to identify opportunities to improve bone health education. Direct-to-patient outreach was used to recruit adult BC patients in the USA self-reporting a diagnosis of bone metastasis within the past 3 years. Of the 200 patients, 59% experienced at least one SRE prior to survey participation (44% radiation to bone, 29% bone fracture, 17% spinal cord compression, 15% surgery to bone), and 83% were currently receiving a BTA. Awareness of general cancer bone health, protection strategies against SREs, and screening tests were low to moderate. Patients currently not receiving a BTA were least knowledgeable about cancer bone health, with only 40% aware of BTAs as a protective strategy, and only 26% were very or extremely satisfied with the information received from healthcare providers. Sixty-two percent of patients wanted to receive information by more than one mode of communication. Notable gaps in bone health education were observed in bone metastatic BC patients at risk for SREs, suggesting the need for earlier and more effective communication and education strategies to promote appropriate BTA use and better health outcomes.

**Supplementary Information:**

The online version contains supplementary material available at 10.1007/s13187-023-02293-w.

## Introduction

Breast cancer (BC) is the most common malignancy and accounts for 15% of new cancer cases in the USA [1]. Of all newly diagnosed BC cases, 6% are de novo metastatic BC [2]. An estimated 75% of US patients currently living with metastatic BC were initially diagnosed with an earlier stage of the disease [3]. Bone is the most common site of distant metastasis and occurs in all subtypes of the disease [4, 5]. Although less than 4% of newly diagnosed BC cases present with de novo bone metastasis (BM), approximately 70% of patients that die of BC will have evidence of BM [6–11].

Bone metastases, often dominated by osteolytic lesions in BC patients, disrupt normal bone remodeling, resulting in a loss of structural integrity and an increased risk for skeletal-related events (SREs), including pathological fractures, spinal cord compression, radiation to the bone for pain, and surgery to the bone for stabilization [12]. BC patients with BM may be at further increased risk of SREs depending on their cancer treatments and preexisting clinical risk factors [13, 14]. Most patients report bone pain at the time of BM diagnosis, and within a year of BM nearly 40% of BC patients experience a SRE [7, 15]. SREs present a significant health and economic burden as they can severely impair activities of daily living and health-related quality of life (HRQoL) and are associated with increased utilization of healthcare resources and mortality [15–18].

In the USA, two different classes of bone targeting agents (BTAs), bisphosphonates and the receptor activator of nuclear factor-kappa β ligand (RANKL) inhibitor denosumab, are indicated for the prevention of SREs in patients with bone metastases from solid tumors, including BC patients with BM. BTAs improve HRQoL by delaying the progression of bone pain and reducing the incidence of SREs [19, 20]. Guidelines from the National Comprehensive Cancer Network (NCCN) support the use of BTAs in BC patients with BM to reduce the risk of SREs [21]. Despite this evidence, real-word data suggest at least 31% of eligible patients do not receive BTA therapy [15, 22, 23]. Reported reasons for not prescribing a BTA include a recent BM diagnosis, poor prognosis, and perceived low SRE risk; these reasons may be related to supportive care being overlooked or delayed [15].

Most cancer-related bone health studies utilize electronic health record databases and physician opinions to understand real-world treatment of cancer patients at elevated risk for SREs, with few studies leveraging the insights directly from patients [15, 24–26]. Leveraging a unique direct-to-patient recruitment approach, we previously conducted the BonE heAlth eduCatiOn Needs assessment (BEACON) survey among patients with multiple myeloma or BM secondary to solid tumors to understand sources, types, and extent of bone health education, and to identify opportunities to improve cancer-related bone health education to enhance appropriate BTA use and health outcomes [26]. In this study, we exclusively expanded recruitment of BC patients with BM to focus on bone health education practices and knowledge.

## Methods

### Study Design

An online cross-sectional survey study was conducted in US BC patients with BM to evaluate awareness of cancer-related bone health. The BEACON survey contained questions about patient demographics; timing of diagnosis; cancer treatments received; experience with cancer-related SREs; current use of a BTA; awareness of general cancer-related bone health, bone health protection, and screening for bone health; and bone health education received (source(s), mode(s), amount, and level of satisfaction). Detailed information about the study design and survey content was previously published [26]. The study was approved by Western Institutional Review Board and conducted in accordance with the Declaration of Helsinki.

### Study Population

A unique direct-to-patient recruitment approach was used, which involved the GRYT Health Cancer Community, cancer non-profit organizations and support groups, patient-targeted outreach via social media platforms, paid targeted online advertising, and GRYT Health’s annual Global Virtual Cancer Conference. Eligible adult patients resided in the USA and were diagnosed with self-reported BM from BC within 3 years of study recruitment. All patients provided e-consent and were screened prior to participation in the BEACON survey. Patients received compensation following survey completion. GRYT Health managed all patient interactions. Study data included 74 BC patients with BM from the initial BEACON survey study population [26]. All data reported is based on patient response; medical records were not requested or reviewed.

### Data Analysis

All analyses are descriptive, and the results are presented as frequency and percentage. For analysis, patient data were categorized based on report of a cancer-related SRE prior to survey participation and current use of a BTA.

## Results

### Patient Characteristics

A total of 200 BC patients with BM completed the BEACON survey. Patient characteristics are summarized in Table 1. Patient age ranged between 26 and 72 years, with a median age of 45 years, and nearly all were female (*n* = 199). Thirty-six percent of patients were in the south, the most populous US census region, and the remaining patients were evenly distributed among the remaining three regions (21–22% each). Sixty-nine percent of patients had a college or higher degree. Private insurance (77%) was the most common medical insurance coverage. Distribution of demographics remained consistent when the population was stratified by reported SRE or current BTA utilization status.

**Table 1:** Patient demographic and clinical characteristics

Characteristic	All patients (*n* = 200)	SRE		Currently receiving a BTA	
		No (*n* = 82)	Yes (*n* = 118)	No (*n* = 35)	Yes (*n* = 165)
Sex, *n* (%)					
Female	199 (99.5)	81 (98.8)	118 (100.0)	35 (100.0)	164 (99.4)
Male	1 (0.5)	1 (1.2)	0 (0.0)	0 (0.0)	1 (0.6)
Age, years					
Mean ± SD	46.6 ± 10.6	45.2 ± 10.1	47.5 ± 10.9	47.5 ± 9.1	46.4 ± 10.9
Range	26–72	28–69	26–72	34–68	26–72
US region, *n* (%)					
Northeast	44 (22.0)	18 (22.0)	26 (22.0)	8 (22.9)	36 (21.8)
Midwest	43 (21.5)	17 (20.7)	26 (22.0)	7 (20.0)	36 (21.8)
South^a^	71 (35.5)	30 (36.6)	41 (34.7)	13 (37.1)	58 (35.2)
West	42 (21.0)	17 (20.7)	25 (21.2)	7 (20.0)	35 (21.2)
Highest level of education, *n* (%)					
High school or GED	15 (7.5)	7 (8.5)	8 (6.8)	3 (8.6)	12 (7.3)
Some college or post-high school education/training	47 (23.5)	15 (18.3)	32 (27.1)	9 (25.7)	38 (23.0)
College degree	78 (39.0)	33 (40.2)	45 (38.1)	13 (37.1)	65 (39.4)
Graduate degree	60 (30.0)	27 (32.9)	33 (28.0)	10 (28.6)	50 (30.3)
Medical insurance^b^, *n* (%)					
Private	153 (76.5)	62 (75.6)	91 (77.1)	24 (68.6)	129 (78.2)
Medicare	32 (16.0)	10 (12.2)	22 (18.6)	5 (14.3)	27 (16.4)
Medicaid	31 (15.5)	13 (15.9)	18 (15.3)	7 (20.0)	24 (14.5)
Other: TRICARE, Veterans Affairs	2 (1.0)	0 (0.0)	2 (1.7)	1 (2.9)	1 (0.6)
None	1 (0.5)	1 (1.2)	0 (0.0)	1 (2.9)	0 (0.0)
Time since diagnosis, *n* (%)					
< 1 year	29 (14.5)	11 (13.4)	18 (15.3)	8 (22.9)	21 (12.7)
1 year	32 (16.0)	16 (19.5)	16 (13.6)	1 (2.9)	31 (18.8)
2 years	64 (32.0)	27 (32.9)	37 (31.4)	9 (25.7)	55 (33.3)
3 years	75 (37.5)	28 (34.1)	47 (39.8)	17 (48.6)	58 (35.2)
Time of bone metastasis diagnosis, *n* (%)					
Same time as initial cancer diagnosis	73 (36.5)	40 (48.8)	33 (28.0)	14 (40.0)	59 (35.8)
After initial cancer diagnosis	127 (63.5)	42 (51.2)	85 (72.0)	21 (60.0)	106 (64.2)
Treatment types received^b^, *n* (%)					
Chemotherapy	148 (74.0)	60 (73.2)	88 (74.6)	26 (74.3)	122 (73.9)
Hormone therapy	166 (83.0)	69 (84.1)	97 (82.2)	23 (65.7)	143 (86.7)
Radiation	131 (65.5)	31 (37.8)	100 (84.7)	18 (51.4)	113 (68.5)
Surgery	131 (65.5)	48 (58.5)	83 (70.3)	21 (60.0)	110 (66.7)

*BTA*, bone targeting agent; *GED*, general educational development; *SD*, standard deviation; *SRE*, skeletal-related event

^a^Includes US territories

^b^Denotes that more than one response could be selected

Seventy percent of patients had been diagnosed with BC at least 2 years prior to completing the BEACON survey. For 37% of patients, the incident BC diagnosis also included a BM diagnosis, while BM reportedly occurred following disease progression or recurrence in the remaining patients. Hormone therapy (83%) was the most common treatment received. Of the types of treatment (chemotherapy, hormone therapy, radiation, and surgery) that patients were asked if they had received, 68% had received at least three of the four types of treatment. Patients could also write in other treatment types, such as targeted therapy. Eighty-three percent of patients were currently receiving a BTA, of which 58% were receiving denosumab and 42% were receiving a bisphosphonate.

### Cancer-Related SREs

Cancer-related SREs prior to survey participation, including bone fracture, spinal cord compression, and radiation and/or surgery to the bone, were reported by 59% of patients and are presented in Online Resource 1. Surgery to the bone (15%) was the least reported type of SRE among all patients, while radiation to the bone was the most frequently reported SRE type (44%). Of the patients with a prior SRE, 31% had experienced only one SRE, 35% had experienced two or three SREs, and 34% had experienced four or more SREs. At least four radiation events and one spinal cord compression were reported by 40% and 45%, respectively, of patients reporting a SRE and currently not receiving a BTA. Meanwhile, of patients with a reported SRE that were currently receiving a BTA, 16% had experienced at least four radiation events and 26% had experienced at least one spinal cord compression.

### Awareness of Bone Health

Patients responded to bone health statements in the survey based on the information they received from their healthcare providers (HCPs) (Table 2). When presented with general bone health statements, more than half of patients were aware of the availability of treatments to protect bone health (68%) and the effect of cancer on the bone (up to 64%), but patients had less awareness of the effect of lifestyle changes (40%) and cancer treatment (radiation, 33%; chemotherapy, 27%) on bone health. Sixty percent of patients not currently receiving a BTA were unaware that treatments are available to protect bone health and 26% of those currently receiving a BTA were unaware. Even in patients that reported receiving radiation therapy and chemotherapy, awareness of the impact of radiation and chemotherapy on bone health remained low (44% and 31%, respectively). Thirty-eight percent of all patients were aware of more than half of the general statements presented. For all general statements, awareness decreased in patients that were SRE naïve or that were not currently receiving a BTA; however, this decrease was more pronounced in the group not receiving a BTA. Twelve percent of all patients were aware of none of the general statements; the lack of awareness was exacerbated in the group not currently receiving a BTA (31%).

**Table 2:** Bone health information shared or recommendations made by HCPs

Bone health statement	All patients (*n* = 200)	SRE		Currently receiving a BTA	
		No (*n* = 82)	Yes (*n* = 118)	No (*n* = 35)	Yes (*n* = 165)
General bone health^a^, *n* (%)					
Bones are more fragile in individuals who have cancer	128 (64.0)	44 (53.7)	84 (71.2)	13 (37.1)	115 (69.7)
Bones are more fragile in individuals who have received radiation therapy	65 (32.5)	12 (14.6)	53 (44.9)	6 (17.1)	59 (35.8)
Bones are more fragile in individuals who have received chemotherapy	53 (26.5)	18 (22.0)	35 (29.7)	4 (11.4)	49 (29.7)
Individuals with cancer have a greater risk of experiencing broken bones because of their cancer	118 (59.0)	40 (48.8)	78 (66.1)	11 (31.4)	107 (64.8)
There are lifestyle changes individuals can make to help prevent broken bones caused by cancer	80 (40.0)	29 (35.4)	51 (43.2)	9 (25.7)	71 (43.0)
There are treatments available to help prevent broken bones caused by cancer	136 (68.0)	53 (64.6)	83 (70.3)	14 (40.0)	122 (73.9)
None of the above; HCPs have not discussed bone health	24 (12.0)	12 (14.6)	12 (10.2)	11 (31.4)	13 (7.9)
Bone health protection strategies^a^, *n* (%)					
Use of calcium and/or vitamin D supplements	155 (77.5)	62 (75.6)	93 (78.8)	22 (62.9)	133 (80.6)
Regular physical activity and weight-bearing exercises	104 (52.0)	44 (53.7)	60 (50.8)	13 (37.1)	91 (55.2)
Lifestyle changes (e.g., stopping smoking or reducing alcohol consumption)	43 (21.5)	16 (19.5)	27 (22.9)	6 (17.1)	37 (22.4)
Treatment with BTAs	156 (78.0)	64 (78.0)	92 (78.0)	14 (40.0)	142 (86.1)
None of the above; HCPs have not discussed preventative strategies	7 (3.5)	3 (3.7)	4 (3.3)	5 (14.3)	2 (1.2)
Bone health screening tests^a^, *n* (%)					
Screening test for osteoporosis or evaluation of bone mineral density	71 (35.5)	24 (29.3)	47 (39.8)	12 (34.3)	59 (35.8)
Blood test to check calcium and/or vitamin D levels	138 (69.0)	57 (69.5)	81 (68.6)	16 (45.7)	122 (73.9)
Fracture risk assessment	14 (7.0)	3 (3.7)	11 (9.3)	2 (5.7)	12 (7.3)
Not applicable; HCPs have not recommended screening tests	50 (25.0)	23 (28.0)	27 (22.9)	15 (42.9)	35 (21.2)

*BTA*, bone targeting agent; *HCP*, healthcare provider; *SRE*, skeletal-related event

^a^Denotes that more than one response could be selected

Awareness of strategies for bone health protection and screening recommendations were also examined (Table 2). Ninety-seven percent of all patients were aware of at least one bone health protection strategy. Patients were aware of calcium and/or vitamin D supplements (78%) and BTAs (78%) for bone health protection, but there was less awareness around physical activity (52%) and lifestyle changes (22%). SRE status did not impact awareness of protection strategies. For all bone health protection statements, awareness was decreased in the group not currently receiving a BTA; however, the decrease was particularly notable for awareness of BTAs (40% in contrast to 86% in patients currently receiving a BTA). While 4% of all patients were unaware of any of the protection strategies, this percentage increased to 14% in the patients not currently receiving a BTA. Seventy-five percent of patients were recommended at least one bone health screening test. Of the screening tests presented, patients were most commonly recommended a test to check blood calcium and/or vitamin D levels (69%); patients not currently receiving a BTA were less likely to be recommended this test (46%). Forty-three percent of patients not currently receiving a BTA were not recommended any screening tests.

### Receiving Bone Health Information

**Fig. 1:**
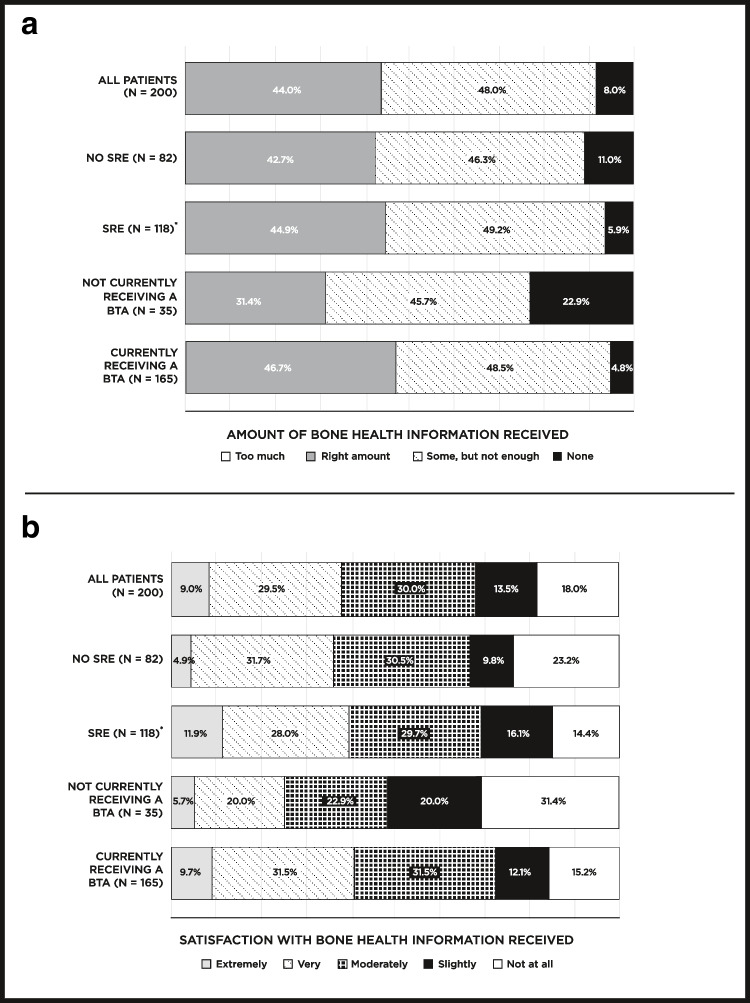
The **a** amount of and **b** satisfaction with bone health information received by patients. Data are also stratified based on prior skeletal-related event (SRE) status and bone targeting agent (BTA) utilization status at the time of survey completion. *Timing of SRE(s) in relation to BTA use is unknown

When asked which HCPs provided information on bone health, patients commonly reported oncologists (96%) followed by nurse practitioners (52%) and radiologists (48%). Fewer than a third of patients received any bone health information from physical therapists (28%), pharmacists (22%), patient navigators (19%), dieticians or nutritionists (16%), or social workers (10%). Patients could also list other HCPs who had provided cancer-related bone health information; despite the survey using the term “radiologist,” rather than “radiation oncologist,” no patients further specified that information was received from a radiation oncologist. Among all patients, 88% reported that information about bone health was shared through discussion(s) with their HCP(s) (Online Resource 2). Only a small proportion of patients reported receiving bone health information through other modes of communication (paper handout, 14%; email, 3%; video, 1%). When patients were asked the preferred mode(s) of communication for bone health information, such as in discussions with their HCP(s) and in printed or electronic materials, 62% indicated more than one mode of communication would be preferred. Eighty-nine percent indicated a desire for discussions about bone health with their HCP(s), but patients also indicated a desire to have the information made available in other forms, most commonly as a physical handout (56%). No one reported receiving too much bone health information, and 56% of patients reported receiving either not enough or no information (Fig. 1). Only 39% were very or extremely satisfied with the amount of bone health information they received. Patients not currently receiving a BTA were both most likely to report not receiving information (23%) and were not at all or only slightly satisfied with the information received (51%).

## Discussion

Multiple factors contribute to bone loss in BC patients, ranging from pre-existing clinical factors such as advanced age, low bone mineral density, and personal or family history to treatments received such as hormone therapy, chemotherapy, and radiation [13, 14]. Metastasis to the bone causes additional changes within the bone that compromise its structural integrity. Because of these converging impacts on the bone and as advancements in treatment extend the life of BC patients with BM, preserving bone health in these patients is critical to delaying and preventing painful SREs and maintaining HRQoL. This study examined patient experience with and awareness of bone health to understand gaps in patient bone health education.

Through the examination of electronic health care records and physician surveys, earlier studies have focused on BM and SRE incidence, impacts of SREs on HRQoL, and utilization and frequency of BTA therapy; however, few studies have directly engaged patients at risk for SREs for their experience, awareness, perception, and/or goals around bone health. Cancer-related bone health studies that have engaged patients have recruited them through the patients’ treatment center and their HCPs [15, 24, 25]. In this study, patient recruitment was achieved primarily through the GRYT Health Cancer Community and its relationships within the cancer community. While this approach did not allow for verification of patients’ health records or the ability to match data to that of their HCP, it did prevent potential recruitment bias by a patient’s HCP and/or treatment center, which allowed for the real-world assessment of patient knowledge and retention of information about bone health.

Study findings indicated that general bone health awareness was low to moderate. Although the majority of patients were familiar with the impact of cancer on bone health and that treatments are available to protect bone health, 32–41% of all patients were unfamiliar with this information. This study did not investigate why a subset of patients currently receiving a BTA reported that their HCP(s) did not make them aware that treatments are available to protect bone health; however, this highlights a gap in education. Awareness of the impact of cancer treatments, such as radiation and chemotherapy, on bone health was low (27–33%) and remained low (< 45%) in patients that received those treatments, suggesting a need for more tailored education and awareness to this specific area. How and when information was shared as well as if information was shared more than once may impact recall. Although the impact of patient recall bias on these responses is not known, to help explain the potential bias influence, this study stratified patient responses by both report of a SRE and current BTA use.

Management of bone health includes both lifestyle recommendations and pharmacological intervention. More than 75% of patients were aware of pharmacologic interventions such as calcium and/or vitamin D supplements and BTAs as a preventative strategy for bone health; however, awareness of regular exercise and lifestyle changes as preventative strategies was lacking. Awareness responses were comparable to previous observations in a mixed cancer patient cohort at risk for SREs [26]. Approximately 70% of patients responded via an open response on what they wished they had been told about the health of their bones; many still had unanswered questions. Respondents wanted to know about bone physiology and how disease affected it, how to monitor bone health, SRE statistics, supplements and BTAs, and lifestyle recommendations, commonly around diet and exercise.

Bone health information was frequently communicated to patients in a discussion with their oncologist. Compared to earlier data in a mixed cancer patient cohort, more patients were receiving information from the radiologist involved in their cancer care; this may reflect more BC patients receiving radiation therapy as part of their treatment [26]. Bone metastatic BC patients may also receive radiation prior to experiencing a SRE as ongoing studies are investigating the role of stereotactic ablative radiotherapy (SABR) in BC patients with oligometastatic disease to improve overall survival and progression-free survival [27, 28]. Regardless, these data emphasize the importance of the conversation with the oncologist and suggest that the knowledge of other HCPs is underutilized. Study findings indicated that patients are generally not satisfied with the amount of bone health information they are receiving and expressed an interest in other communication modes, such as printed or electronic materials, to supplement bone health discussions with HCPs.

Patient data were also analyzed based on report of a prior SRE and current BTA status. Although the percentage of SRE naïve patients was influenced by study design and is not reflective of real-world prevalence of SREs, it is relevant to note that 41% of all patients were SRE naïve, and that this percentage was similar in patients not currently receiving (43%) and receiving (41%) a BTA. There was a slight decline in awareness of the general bone health statements in patients that were SRE naïve; however, the greatest deficiency in bone health awareness was in patients that were not currently receiving a BTA, even though these patients were more likely to report more spinal cord compression events and at least four radiation events. Patients not currently receiving a BTA were also less likely to receive or be satisfied with the bone health information from their HCPs.

Current BTA use in this study was higher than other real-world reports [15, 22, 23]. Although 83% of patients were currently receiving a BTA, of which 59% reported an SRE, this study does not suggest low efficacy of BTAs or inappropriate use of BTAs but may be a reflection of the recruiting methodology. Additionally, timing of BTA therapy, specifically in relation to when an SRE was experienced, was not investigated in this study. Patients often decide to start BTA therapy because they are experiencing bone pain, have an elevated risk of SREs, have a history of SREs, or have multiple metastases to the bone [15]. Meanwhile, the factors influencing the BTA decision from physicians are the long-term safety record of BTAs, efficacy in delaying onset of SREs, and efficacy in reducing the risk and/or number of SREs [15].

Of the patients in the group not currently receiving a BTA at the time of survey completion, it is unknown how many of these patients were BTA naïve or how many had received BTA therapy in the past. These patients were also not asked the reason for not currently receiving a BTA at the time of survey completion; however, possible reasons include a recent BM diagnosis, poor prognosis, short life expectancy, patient refusal, a perceived low risk of SREs, and an interruption in BTA therapy [15]. Although this study focused on bone metastatic BC patients and BTA use at the time of survey completion, clinical trials have investigated adjuvant bisphosphonate use in post-menopausal early-stage BC patients; however, a limitation of this study is that it did not capture a prior history of BTA use in a non-metastatic setting [29].

There are limitations that should be considered when interpreting this study. As previously discussed, recall bias may influence the findings of this patient-based study. Additionally, the willingness of a patient to participate in this survey study may be influenced by overall health status and survivor bias, and, thus, cancer severity or metastatic status and treatment may influence the results. The study may not be reflective of the knowledge and/or experience of the average bone metastatic BC population. Study demographics showed that patients were notably young, well-educated, and covered by private insurance. Digital recruitment methods favor patients with access to the internet and that are seeking digital information and/or connections. These patients may be more engaged and/or informed about their health compared to the average patient with a similar diagnosis.

## Conclusions

Gaps in bone health awareness and education delivery were observed in bone metastatic BC patients. These gaps were amplified in patients not receiving current BTA therapy. In particular, patients reported receiving inadequate information about bone-related side effects from cancer treatments, availability of medication(s) to prevent SREs, and information regarding lifestyle changes that may help to protect and preserve bone health. There are opportunities for HCPs to provide more and improved bone health information to patients and for information to be communicated through repeated discussions and varied methods. Patient feedback revealed that the survey unintentionally served as an educational tool, which should be considered in future survey design. There is a need for earlier and more effective patient- and provider-oriented interventions to delay or prevent painful SREs and improve HRQoL, particularly as advancements in treatment extend life expectancy.

### Supplementary information

ESM 1. (DOCX 292 kb)

ESM 2. (DOCX 16 kb)

## Data Availability

Data are available upon reasonable request to the corresponding author.
