# Supplementary material for: Assessment of Bone Health Awareness and Education in Breast Cancer Patients with Bone Metastasis in the USA
Source: J Cancer Educ. 2023 Apr 28;38(5):1522–30. doi: 10.1007/s13187-023-02293-w (PMC10509072; doi:10.1007/s13187-023-02293-w)
Supplement: Supplementary file 1 — (DOCX 292 kb) [file 13187_2023_2293_MOESM1_ESM.docx]

**Journal of Cancer Education**

**Original Article**

**Assessment of Bone Health Awareness and Education in Breast Cancer Patients with Bone Metastasis in the USA**

**Online Resource 1**


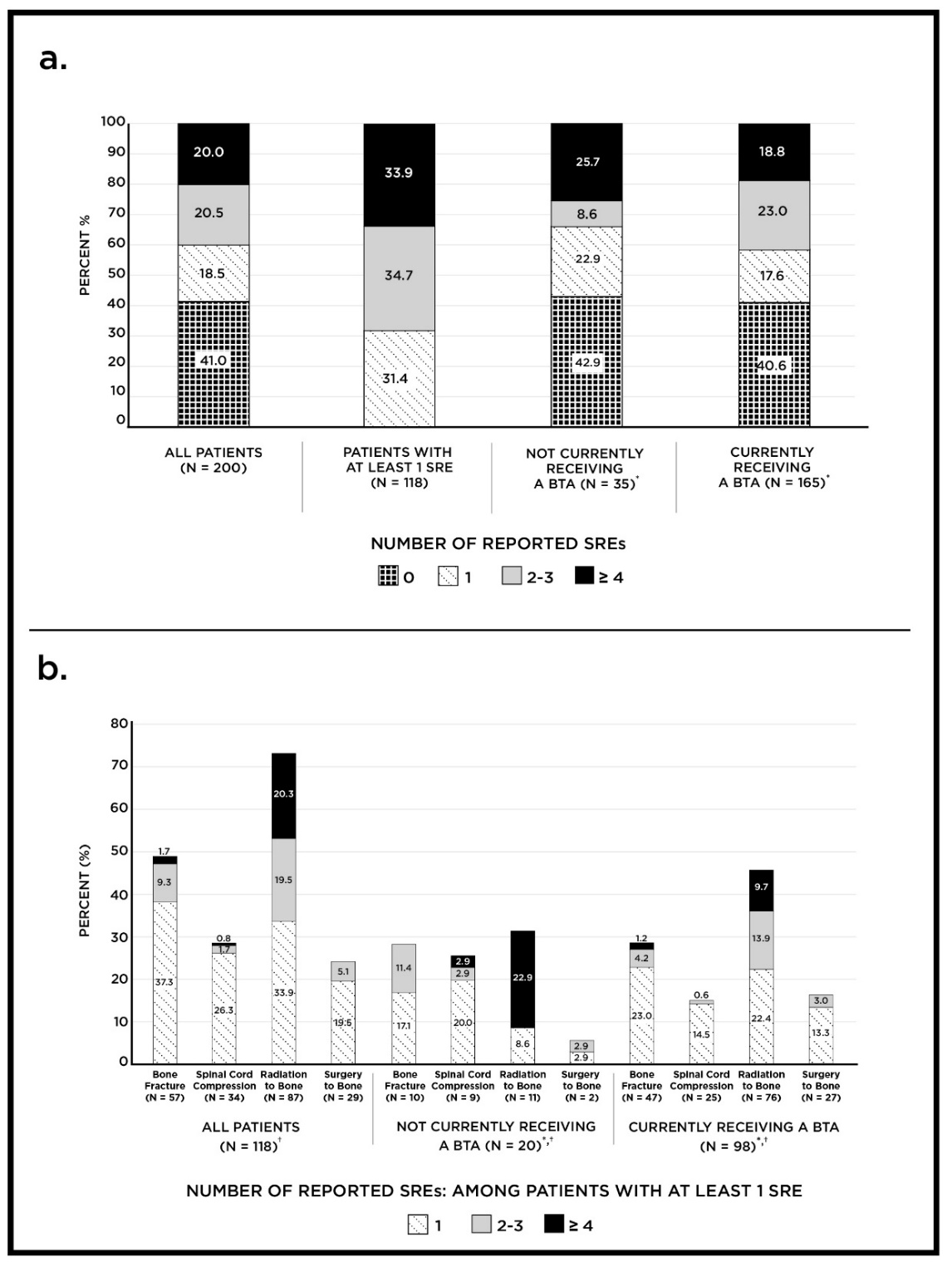


**Online Resource 1** Number of reported skeletal-related events (SREs), including bone fracture, spinal cord compression, radiation to the bone, and surgery to the bone, for (a) all types of SREs among both patients not reporting and reporting a prior cancer-related SRE and (b) each type of SRE among only patients that reported at least one prior cancer-related SRE. Data are also stratified based on the bone targeting agent (BTA) utilization status at the time of survey completion. *Timing of SRE(s) in relation to BTA use is unknown. ^†^Patients could report more than one SRE type
