# Supplementary material for: Assessment of Bone Health Awareness and Education in Breast Cancer Patients with Bone Metastasis in the USA
Source: J Cancer Educ. 2023 Apr 28;38(5):1522–30. doi: 10.1007/s13187-023-02293-w (PMC10509072; doi:10.1007/s13187-023-02293-w)
Supplement: Supplementary file 2 — (DOCX 16 kb) [file 13187_2023_2293_MOESM2_ESM.docx]

**Journal of Cancer Education**

**Original Article**

**Assessment of Bone Health Awareness and Education in Breast Cancer Patients with Bone Metastasis in the USA**

**Online Resource 2**

**Online Resource 2** Sharing of bone health information

|  | | All patients  (*n* = 200) | SRE | | Currently receiving a BTA | |
| --- | --- | --- | --- | --- | --- | --- |
|  |  |  | No  (*n* = 82) | Yes  (*n* = 118) | No  (*n* = 35) | Yes  (*n* = 165) |
| How HCPs share bone health information with patients^a^, *n* (%) | | | | | | |
|  | Discussion | 176 (88.0) | 71 (86.6) | 105 (89.0) | 31 (88.6) | 145 (87.9) |
|  | Paper handout | 28 (14.0) | 10 (12.2) | 18 (15.3) | 1 (2.9) | 27 (16.4) |
|  | Email | 5 (2.5) | 3 (3.7) | 2 (1.7) | 1 (2.9) | 4 (2.4) |
|  | Video | 2 (1.0) | 0 (0.0) | 2 (1.7) | 1 (2.9) | 1 (0.6) |
|  | Other | 1 (0.5) | 0 (0.0) | 1 (0.8) | 0 (0.0) | 1 (0.6) |
|  | No information shared | 21 (10.5) | 9 (11.0) | 12 (10.2) | 4 (11.4) | 17 (10.3) |
| How patients want bone health information shared by HCPs^a^, *n* (%) | | | | | | |
|  | Discussion | 177 (88.5) | 75 (91.5) | 102 (86.4) | 30 (85.7) | 147 (89.1) |
|  | Paper handout | 111 (55.5) | 41 (50.0) | 70 (59.3) | 23 (65.7) | 88 (53.3) |
|  | Email | 62 (31.0) | 21 (25.6) | 41 (34.7) | 11 (31.4) | 51 (30.9) |
|  | Video | 39 (19.5) | 15 (18.3) | 24 (20.3) | 8 (22.9) | 31 (18.8) |
|  | Other | 2 (1.0) | 1 (1.2) | 1 (0.8) | 0 (0.0) | 2 (1.2) |

*BTA*, bone targeting agent; *HCP*, healthcare provider; *SRE*, skeletal-related event

^a^ Denotes that more than one response could be selected
